# The Application of Terahertz Time-Domain Spectroscopy to Identification of Potato Late Blight and Fusariosis

**DOI:** 10.3390/pathogens10101336

**Published:** 2021-10-16

**Authors:** Nikita V. Penkov, Mikhail V. Goltyaev, Maxim E. Astashev, Dmitry A. Serov, Maxim N. Moskovskiy, Dmitriy O. Khort, Sergey V. Gudkov

**Affiliations:** 1 Institute of Cell Biophysics RAS, Federal Research Center “Pushchino Scientific Center for Biological Research of the Russian Academy of Sciences”, 142290 Pushchino, Russia; nvpenkov@rambler.ru (N.V.P.); goltayev@mail.ru (M.V.G.); dmitriy_serov_91@mail.ru (D.A.S.); 2 Prokhorov General Physics Institute of the Russian Academy of Sciences, 119991 Moscow, Russia; astashev.max@gmail.com; 3 Federal State Budgetary Scientific Institution “Federal Scientific Agroengineering Center VIM”, 109428 Moscow, Russia; maxmoskovsky74@yandex.ru (M.N.M.); dmitriyhort@mail.ru (D.O.K.)

**Keywords:** terahertz time-domain spectroscopy, food safety, potato late blight, fusariosis

## Abstract

Fusarium and late blight (fungal diseases of cereals and potatoes) are among the main causes of crop loss worldwide. A key element of success in the fight against phytopathogens is the timely identification of infected plants and seeds. That is why the development of new methods for identifying phytopathogens is a priority for agriculture. The terahertz time-domain spectroscopy (THz-TDS) is a promising method for assessing the quality of materials. For the first time, we used THz-TDS for assessing the infection of seeds of cereals (oats, wheat and barley) with fusarium and potato tubers of different varieties (Nadezhda and Meteor) with late blight. We evaluated the refractive index, absorption coefficient and complex dielectric permittivity in healthy and infected plants. The presence of phytopathogens on seeds was confirmed by microscopy and PCR. It is shown, that Late blight significantly affected all the studied spectral characteristics. The nature of the changes depended on the variety of the analyzed plants and the localization of the analyzed tissue relative to the focus of infection. Fusarium also significantly affected all the studied spectral characteristics. It was found that THz-TDS method allows you to clearly establish the presence or absence of a phytopathogens, in the case of late blight, to assess the degree and depth of damage to plant tissues.

## 1. Introduction

Climate change contributes to the spread of pathogens of agricultural crops throughout the Earth, including diseases caused by fungi of the genera *Phytophthora* and *Fusarium* [1,2]. The main drivers of climate change that can affect the severity and spread of plant diseases are: increased atmospheric CO_2_, heavy and off-season rainfall, high humidity, droughts, cyclones and hurricanes, and higher winter temperatures [3,4,5,6,7]. Plant diseases caused by members of the oomycot family *Phytophthora* are associated with wilting, chlorosis, root rot, and rot of tubers and fruits. Currently, several dozen plant diseases caused by fungi of the genus Phytophthora have been described [8]. A member of the *Oomycota* family *Phytophthora infestans* L. is the most common pathogen of the potato (*Solanum tuberosum* L.), causing late blight (potato late blight) and capable of destroying a significant part of the crop. The most famous consequence of late blight was the Irish Potato Famine in the 1840s [8,9,10,11]. The total damage from late blight exceeds USD $ 6.2 billion per year due to crop losses and the need to use fungicides [12,13]. 

*Fusarium* fungi cause diseases in a wide range of food plants around the world, including wheat, barley, rye, oats, corn, soybeans, onions, cabbage and asparagus [14,15,16,17,18,19,20]. Representatives of the Fusarium genus not only cause enormous damage to the world economy, but also contaminate the raw materials used to create food with mycotoxins (deoxynivalenol, zearalenone, etc.), which in turn already poses a danger to the life and health of humans and animals [21,22]. 

Modern methods for the detection of pathogens predominantly are based on the PRC-analysis [23,24,25]. Despite the prevalence of PCR analysis, it has a number of significant drawbacks. The disadvantages of PCR analysis include the search for genes specific for the studied phytopathogen, the duration of the analysis, and the high cost. Due to the shortcomings of PCR analysis, it becomes necessary to develop a fast and inexpensive method for determining the contamination of plant material with pathogens of the genera *Fusarium* and *Phytophthora*.

In the last decade, the terahertz spectroscopy method has demonstrated high information content in a wide range of applications: material analysis, monitoring of various technological processes, biomedicine, food chemistry and agriculture to determine the nutritional value of seeds [26,27,28,29,30,31,32,33,34,35,36,37,38,39]. It should be noted that THz spectra are most informative when analyzed substances in crystalline form. The point is that narrow, highly characteristic bands in the THz region can be recorded only in the presence of a long-range symmetry order, which is realized in crystals. When analyzing multicomponent samples, especially those with a high content of amorphous and unstructured phases, the spectra lose their characteristic bands [40]. The amorphous phase does not show narrow spectral bands; instead, a broad boson peak is recorded. Note that until now a full-fledged theoretical substantiation of the boson peak and the relationship of its characteristics with the structure of the object under study has not been obtained [41,42,43]. Nevertheless, the sensitivity of THz spectra to the intermolecular structure makes it possible to detect differences even in substances with complex composition and intermolecular structure, and use them for quality control.

The possibility of using the method of THz spectroscopy to assess damage to crops and the possible identification of potato late blight and fusariosis has not been previously studied. THz spectroscopy is almost never used in agriculture. We know of only one single remarkable work on the application of the THz spectroscopy method for the identification of phytopathogens such as fungal infections in chestnuts [44]. The dependence of the parameters of THz spectra on the species and varieties of healthy and affected plants also requires research. In view of the foregoing, the purpose of this work was to assess the effect of late blight or fusarium infection on the THz spectral characteristics of potatoes of different varieties and cereals of different species, respectively. The early stage of late blight is characterized by the defeat of not the entire tuber, but of its individual parts, especially the peel [45,46]. therefore, we additionally investigated the THz spectral characteristics of various tuber tissues. The data obtained will allow in the future to optimize the terahertz spectroscopy technique for assessing the damage to agricultural crops by economically significant plant pathogens.

## 2. Results

### 2.1. Detection of Pathogens and Assessment of the Degree of Contamination of Samples

The infection was verified by RT-PCR. In potato samples “Meteor (+)” and “Nadezhda (+)”, regardless of the presence of a peel, *P. infestans* DNA was identified by real-time PCR (Ct ~ 25). In the samples “Meteor (−)” and “Nadezhda (−)”, regardless of the presence of the peel, *P. infestans* DNA was not identified for 40 cycles. In the samples of Nadezhda “focus”, “0–1” and “1–2”, *P. infestans* DNA was identified. In samples of oats (+), barley (+), and wheat (+), the DNA of *F. avenaceum* (Ct ~ 11) and *F. graminearum* (Ct ~ 14) was identified. In control samples of all cereals (−), DNA of both species was not detected. In each variant of the experiment, three biological samples were analyzed. For each sample, 8 measurements were performed, the results of which were averaged. The proportion of affected seeds was 86, 94 and 98% for oats, barley and wheat, respectively.

### 2.2. Effect of Late Blight on Potato Characteristics Determined by the THz-TDS Method

**Figure 1:**
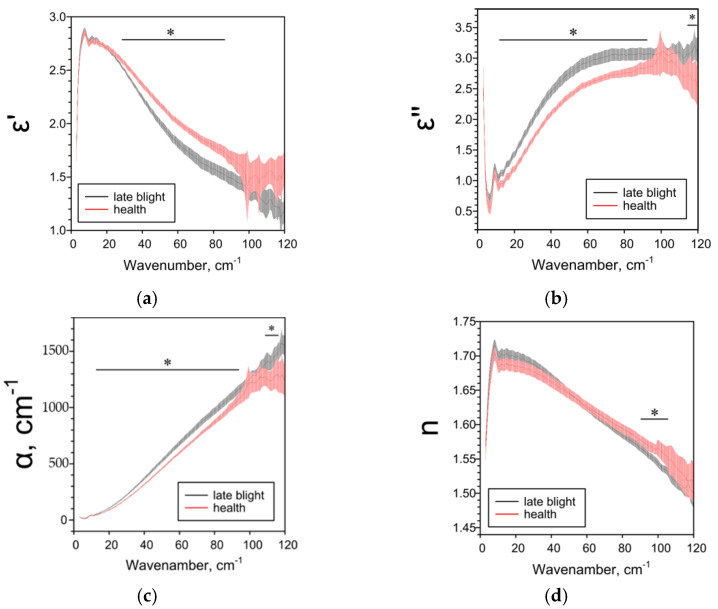
THz-spectral characteristics of whole potato tubers of the “Meteor” variety: (**a**) Real parts of complex dielectric permittivity ε’; (**b**) Imaginary parts of complex dielectric permittivity ε’’; (**c**) The absorption coefficient α; (**d**) Refractive index n. Data are presented as mean ± 95% confidence interval. * *p* < 0.05.

**Figure 2:**
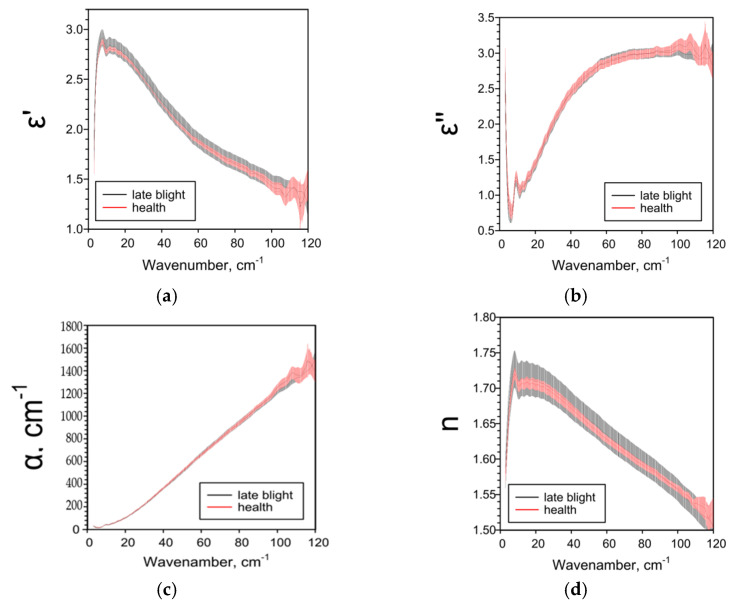
THz-spectral characteristics of potato tubers without peel of the “Meteor” variety: (**a**) Real parts of complex dielectric permittivity ε’; (**b**) Imaginary parts of complex dielectric permittivity ε’’; (**c**) The absorption coefficient α; (**d**) Refractive index n.

**Figure 3:**
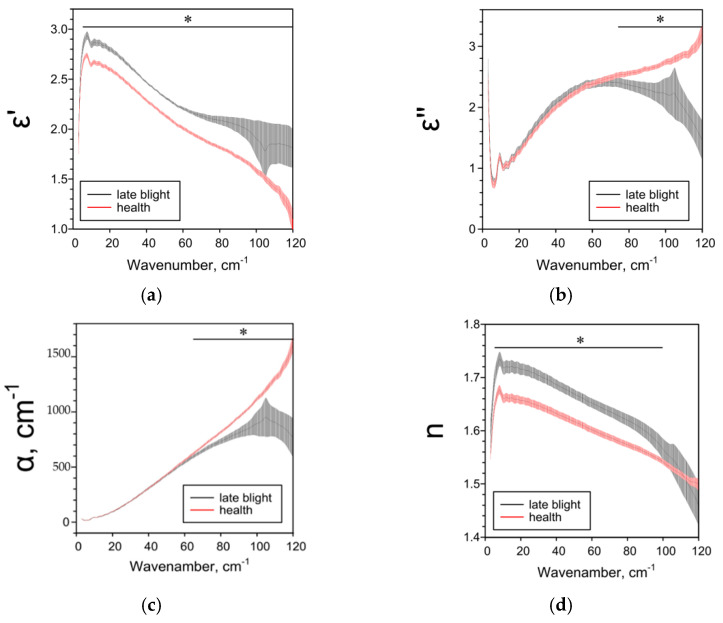
THz-spectral characteristics of whole potato tubers of the “Nadezhda” variety: (**a**) Real parts of complex dielectric permittivity ε’; (**b**) Imaginary parts of complex dielectric permittivity ε’’; (**c**) The absorption coefficient α; (**d**) Refractive index n. Data are presented as mean ± 95% confidence interval. * *p* < 0.05.

**Figure 4:**
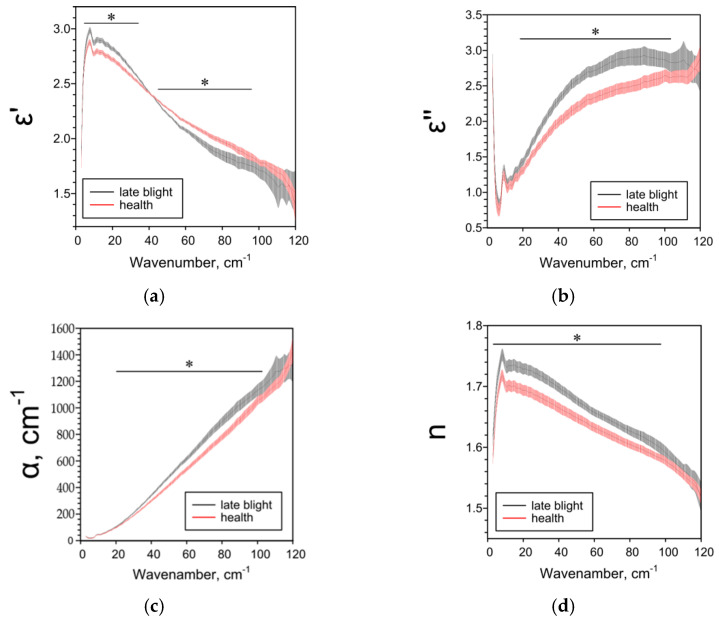
THz-spectral characteristics of tubers without peel of potato variety “Nadezhda”: (**a**) Real parts of complex dielectric permittivity ε’; (**b**) Imaginary parts of complex dielectric permittivity ε’’; (**c**) The absorption coefficient α; (**d**) Refractive index n. Data are presented as mean ± 95% confidence interval. * *p* < 0.05.

Late blight affects all studied THz spectra of potato tubers. THz-TDS results are highly dependent on the potato variety and tissue analyzed. For whole tubers of the more resistant cultivar “Meteor”, when infected with late blight, a decrease in ε’ and n and an increase in ε’’ and α were observed in comparison with healthy tubers (Figure 1). Removing the peel was neutralized the effects of phytophthora (Figure 2). The terahertz spectral characteristics of cultivar “Nadezhda”, less resistant to late blight, significantly differed from those for cultivar “Meteor” (Figure 3). In the case of whole tubers, late blight increased ε’ and n and decreased ε’ and α. The peeled tuber had different characteristics (Figure 4). Late blight changed ε’ in a complex way: it increased in the range of higher frequencies (44–95 cm^−1^) and decreased in the range of lower frequencies (5–38 cm^−1^). The remaining characteristics ε’’, α and n in late blight were increased in wide frequency ranges.

**Figure 5:**
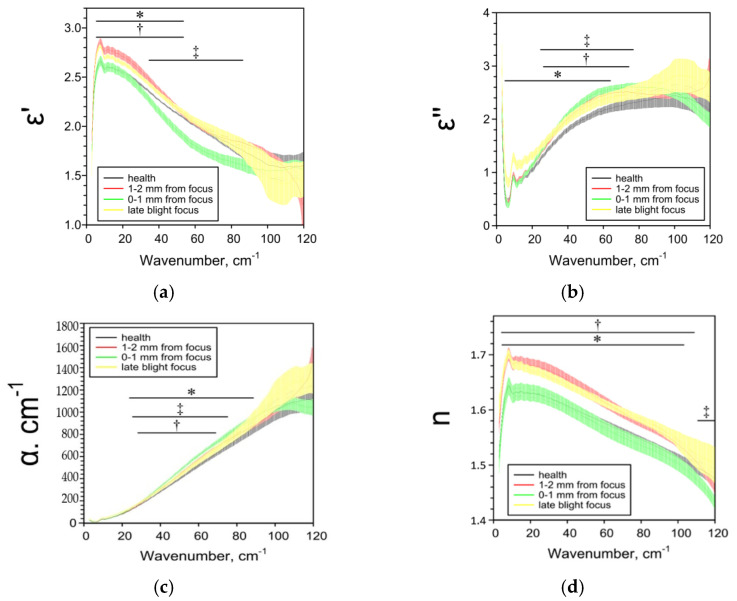
THz-spectral characteristics of sections of the peel of the “Nadezhda” potato at different distances from the center of late blight: (**a**) Real parts of complex dielectric permittivity ε’; (**b**) Imaginary parts of complex dielectric permittivity ε’’; (**c**) The absorption coefficient α; (**d**) Refractive index n. Data are presented as mean ± 95% confidence interval. * *p* < 0.05, health vs. focus; † *p* < 0.05, health vs. 1–2 mm from focus; ‡ *p* < 0.05, health vs. 0–1 mm from focus.

The terahertz spectral characteristics also depend on the localization in the peel relative to the lesion focus of late blight (Figure 5). When approaching the focus, an increase in ε’’, α and n, an increase in ε’ with a subsequent decrease was found.

### 2.3. Effect of Fusarium on the Characteristics of Cereals Determined by the THz-TDS Method

**Figure 6:**
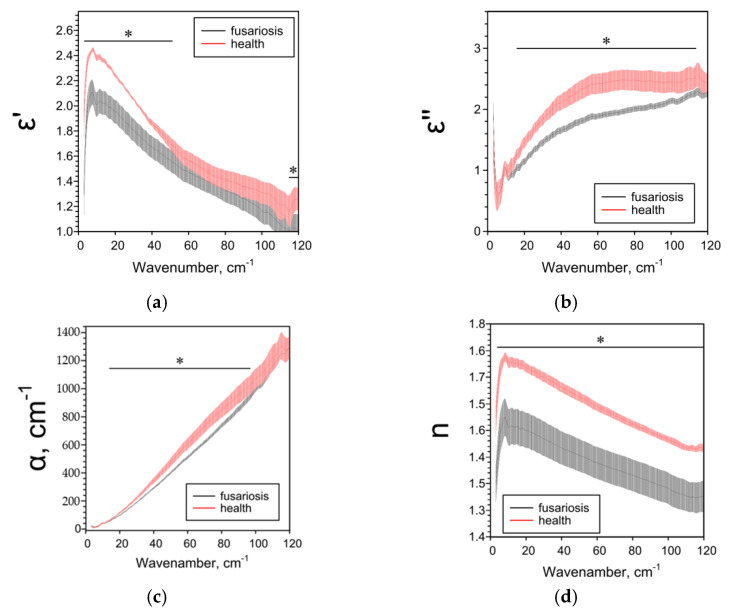
THz-spectral characteristics of oat seeds: (**a**) Real parts of complex dielectric permittivity ε’; (**b**) Imaginary parts of complex dielectric permittivity ε’’; (**c**) The absorption coefficient α; (**d**) Refractive index n. Data are presented as mean ± 95% confidence interval. * *p* < 0.05.

**Figure 7:**
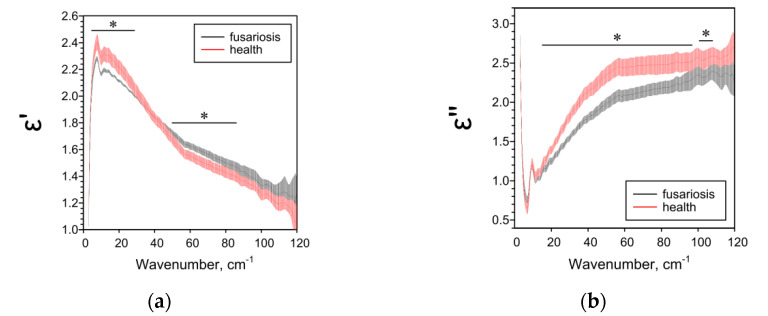

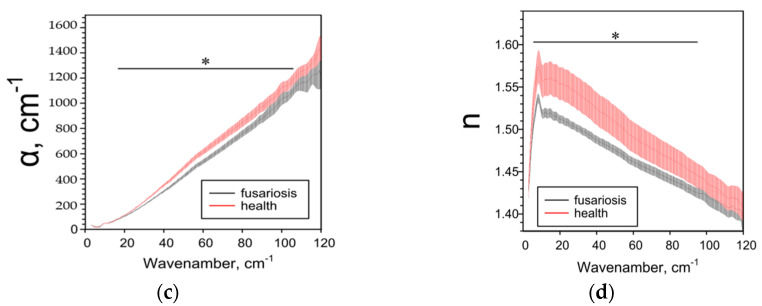
THz spectral characteristics of wheat seeds: (**a**) Real parts of complex dielectric permittivity ε’; (**b**) Imaginary parts of complex dielectric permittivity ε’’; (**c**) The absorption coefficient α; (**d**) Refractive index n. Data are presented as mean ± 95% confidence interval. * *p* < 0.05.

**Figure 8:**
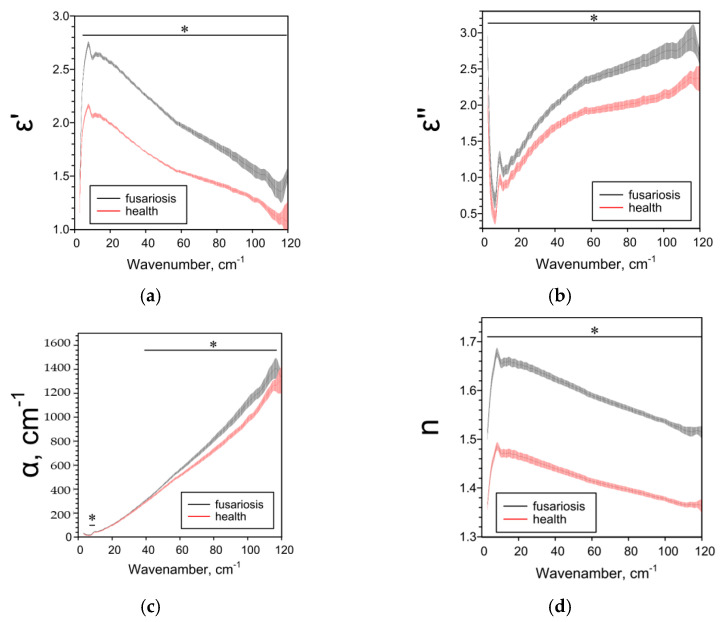
THz spectral characteristics of barley seeds: (**a**) Real parts of complex dielectric permittivity ε’; (**b**) Imaginary parts of complex dielectric permittivity ε’’; (**c**) The absorption coefficient α; (**d**) Refractive index n. Data are presented as mean ± 95% confidence interval. * *p* < 0.05.

The effect of Fusarium on the THz spectral characteristics of seeds significantly depended on the species of the host plant. In the case of oats, Fusarium disease decreased all the studied spectral parameters (Figure 6). In the case of wheat, Fusarium reduced ε’’, α and n over a wide frequency range and had a complex effect on ε’ (Figure 7). With fusarium, ε’ decreased in the low-frequency range of 5–29 cm^−1^ and increased in the high-frequency range of 50–86 cm^−1^. In the case of barley, all the studied parameters in case of fusarium increased practically over the entire analyzed frequency range (Figure 8).

## 3. Discussion

We used the THz-TDS method for the first time to characterize the effect of late blight on absorption (α), refractive index (n) and complex dielectric permittivity (ε’, ε’’) in potatoes. The THz-TDS method allows to clearly establish the presence or absence of the pathogen in the plant, as well as assess the degree and depth of damage to plant tissues. If the variety “Nadezhda” [47] with the skin is considered the most affected, and the variety “Meteor” without the skin is the least, then based on the data obtained and the characteristics of varieties^46^, we can assume the following dependence of the THz spectra of parameters on the degree of late blight. As the phytopathogen spreads: ε’ and n first decrease (early stages), then increase; ε’’ and α first increase, then decrease (Table 1). The data on the dependence of ε’ on the degree of injury are consistent and when analyzing different parts of the peel (Figure 5), near the focus there is a decrease in ε’, but not in the focus itself. All investigated parameters ε’, ε’’, α and *n* are sensitive to weak, strong and moderate lesions by late blight (Figure 1, Figure 2, Figure 3, Figure 4 and Figure 5), therefore they can be used both for screening lesions in the early stages and assessing the degree of tuber damage. We were the first to obtain data on the dependence of THz-spectral characteristics on the degree of damage to potatoes by late blight.

Significant change in the parameters of terahertz spectra during late blight can be caused by two reasons. The first reason is a change in the composition of potato sugars due to the metabolism of the fungus. Thus, late blight manifests itself in the degradation of starchz [48]; therefore, more cellulose remains in the infected samples against the background of decrease in the proportion of starch. The second reason is the growth of the biomass of the fungus itself. The fungi of the *Oomycota* group are characterized by a cellulose cell wall [49]; therefore, the growth of biomass can be associated with an increase in the cellulose content in tubers. As you know, cellulose in different crystalline forms has absorption peaks ranging from 60 to 85 cm^−1^ and from 95 to 110 cm^−1^ [50]. Both reasons lead to increase in the cellulose content in the samples, which increases the absorption in the above frequency bands.

We used the THz-TDS method for the first time to characterize the effect of fusarium on the THz spectral characteristics of seeds of different types of cereals. We found a dependence of the effects of fusarium on THz-spectra on the type of affected cereal. The differences in the effects of fusarium on THz spectral characteristics can be explained by the specific features of the composition of cereal seeds (Table 2). In particular, the literature describes differences in the ratio of soluble and insoluble dietary fiber in seeds: oats have more soluble fibers (SDF), in particular starch, and in wheat and barley, insoluble fibers (IDF), in particular cellulose [50,51,52,53].

The increase in the absorption rate in oat grains infected with *Fusarium* can be explained as follows. Starch has been shown to be a preferred substrate for *Fusarium* over cellulose [54]. For oats with a high starch content (low IDF / SDF ratio), *Fusarium* will reduce starch and increase cellulose. In the case of wheat and barley, the effect will be the opposite. These cereals have a high IDF/SDF ratio, therefore, a higher proportion of cellulose. With a high cellulose content compared to other sugars, it can act as a substrate for *Fusarium* [55]. In this case, *Fusarium* will lead to a decrease in the concentration of cellulose in the seeds. Our data indirectly confirm this assumption. In addition, our data on a decrease in the absorption rate in wheat under *Fusarium* disease coincide with the literature data on a decrease in the absorption rate in seeds affected by mold compared to healthy seeds [56].

## 4. Materials and Methods

### 4.1. Plants Samples

In the study of late blight, two varieties of *Solanum tuberosum* tubers were used: “Meteor” and “Nadezhda”. Late blight was diagnosed organoleptic, in addition, the presence of the pathogen Phytophthora infestans in the tissues of tubers was confirmed by microscopy using Mikmed 2 (Mikmed, Russia). Samples were divided into infected (+) or non-infected (−). For a more accurate determination of the localization of the pathogen in the tuber tissues, samples of potatoes with skin (with K) and without (without K) were studied separately. We also examined samples obtained from different places of the same tuber: the focus of rot, in a cut of 0–1 or 1–2 mm from the focus of rot, denoted hereinafter “focus”, “0–1” and “1–2”, respectively. Potato samples were obtained from the Federal Scientific Agroengineering Center VIM Pathogen Collection. Variety “Meteor” was chosen as more resistant to late blight, and variety “Nadezhda” as moderately resistant [47].

In the study of fusarium, we used samples of oat cereals *Avena sativa*, barley *Hordeum vulgare*, and wheat *Triticum aestivum*, healthy (−) and affected by fusarium (+). The proportion of affected seeds was 86, 94 and 98% for oats, barley and wheat, respectively. Fusarium blight was identified by the presence of the pathogen *Fusarium sp.* by microscopy. Samples were provided by Federal Scientific Agroengineering Center VIM, Moscow.

### 4.2. DNA Extraction

To verify the microscopic data, we performed diagnostics of fusarium and potato scab by real-time PCR. Isolation of genomic DNA from samples was performed using cetyltrimethylammonium bromide (CTAB method). A detailed description of the method was given earlier [57].

### 4.3. Real-Time PCR

Primers specific for these pathogens were used to identify *Fusarium avenaceum*, *Fusarium graminearum* and *Phytophthora infestans* in the respective samples (Table 3). All primers were synthesized at Evrogen (Moscow, Russia). The reaction mixture was prepared by mixing 5 µL of the ready-to-use qPCRmix-HS SYBR mixture (Evrogen, Moscow, Russia) with a pair of target primers (1 µL each), 1 µL of the template DNA solution (1.28 × 10^2^ ng/mL) and Milli-Q water to a volume of 25 µL. The real-time PCR reaction was performed in an O-DTLITE 4S1 amplifier (DNA technology, Russia). PCR for the identification of *P. infestans* was carried out according to the following protocol: denaturation for 5 min at 94 °C, then 40 cycles for 5 s at 94 °C, 30 s at 60 °C and 20 s at 72°C. PCR for verification of fusarium was carried out according to the following protocol: denaturation for 85 s at 94 °C, then 25 cycles 35 s at 95 °C, 30 s at 5 3°C and 30 s at 72° C. Fluorescence intensity measurements were performed at the end of the 72°C cycle. Ct values, standard curves and corresponding correlation coefficients (R^2^) were automatically obtained using Sequence Detection System v.1.2 software (Waltham, MA, USA) by interpolating *C*t values against decimal logarithms of the original DNA concentrations. As a negative control, 2 µL of Milli-Q water was added to the reaction mixture instead of the DNA template. Three independent measurements were performed for each variant.

### 4.4. Plants Sample Preparation to THz-TDS

The plant material was subjected to mechanical grinding followed by drying under vacuum (1 mbar, 1 day). Then the powders were ground using a ball mill until an average particle size of about 10 μm was reached. The largest particles did not exceed 100 μm. The grinding of the samples took place stepwise. Balls of different weights and sizes were used at different stages of grinding. Particle size was confirmed by light microscopy.

Each sample in the amount of 50 mg was mixed with 125 mg Polyethylene powder, 40–48 μm particle size (Sigma-Aldrich, Saint Louis, MO, USA). The mixture was thoroughly ground in an agate mortar to achieve uniform mixing. After that, the mixture was compressed at 5 tons to cylindrical pellet of 13 mm diameter and about 1.4 mm height. In a similar way, a 125 mg pellet of pure polyethylene was prepared to record the background spectrum. For each sample, 3 identical pellets were made.

### 4.5. Terahertz Time-Domain Spectroscopy (THz-TDS)

The obtained pellets with the studied samples were analyzed using the Terahertz time-domain spectroscopy (THz-TDS) method. The THz-TDS method consists in measuring the time profile of the electric field strength E(*t*) of a picosecond pulse. As a result of the complex Fourier transform E(*t*) of pulses passing through the sample and without the sample (background pulse), the transmission *Tr(ν*) and refractive index n (ν) spectra of the sample can be calculated. The complex dielectric permittivity (ε’, ε’’) is uniquely calculated from these two spectra without using the Kramers-Kronig relations. The details of the THz-TDS method are well known and have been described previously [58]. The spectra were measured on a TPS Spectra 3000 spectrometer (Teraview, UK) in the range 3–120 cm^−1^ with a spectral resolution of 2 cm^−1^. Dielectric permittivity was calculated using the following relations:

(1) $$\varepsilon^{\prime }\left(\nu \right)=n^{2}\left(\nu \right)-{\left[\frac{\ln Tr\left(\nu \right)}{4\pi \nu l}\right]}^{2}$$

(2) $$\varepsilon^{\prime \prime }\left(\nu \right)=-\frac{n\left(\nu \right)\ln Tr\left(\nu \right)}{2\pi \nu l}$$

where $\varepsilon^{\prime }$ и $\varepsilon^{\prime \prime }$ are the real and imaginary parts of the dielectric permittivity, *ν* is the wavenumber, *l* is the thickness of the measured sample. The thickness of the sample was considered to be the difference between the thicknesses of a pellet with a sample and a pellet made of pure polyethylene. The thickness of the pellets was measured with a micrometer MK 0–25 mm (Kalibr, Moscow, USSR) with an accuracy of 5 μm. The absorption coefficient spectra were also analyzed:

(3) $$\alpha \left(\nu \right)=-\frac{\ln Tr\left(\nu \right)}{l}$$

After placing each sample in the cell compartment, a pause of 5 min was maintained before the start of measurement to ensure that the optical part of the spectrometer was purged with dry air using an FT-IR Purge Gas Generator 74–5041 (Parker Hannifin Corporation, Haverhill, MA, USA). Purging is necessary to obtain high-quality spectra, since water vapor strongly absorbs in the THz region. Each sample was measured at least 8 times.

### 4.6. Statistical Analysis

Data are presented as mean values ± 95% confidence intervals. The statistical significance of the differences was assessed by the degree of overlapping of the confidence intervals: in the absence of overlaps, the differences between the compared sample means were considered statistically significant.

## 5. Conclusions

For the first time, the THz-TDS method was used to characterize the effect of late blight and fusarium on absorption, refractive index and complex dielectric permittivity in potatoes of different varieties and cereals of different types. The causative agents of the diseases changed all the studied characteristics. At the same time, the nature of the changes depended on the varietal and species belonging of the analyzed plants, as well as the localization of the tissue relative to the focus of infection in the case of late blight. The THz-TDS method allows you to clearly establish the presence or absence of a pathogen in a plant, as well as assess the degree and depth of damage to plant tissues. In the case of cereals, the species must be taken into account for an adequate diagnosis of fungal infection. The THz-TDS method can be considered as a promising approach for mass, rapid and relatively inexpensive screening of agricultural crops for the presence of economically significant plant infections.

## Figures and Tables

**Table 1:** Effect of late blight of different severity on the THz spectral characteristics of tubers.

Parameter	The Extent of the Late Blight			
	Ultra Low	Low	Intermediate	Severe
	“Meteor” without peel	“Meteor” with peel	“Nadezhda” without peel	“Nadezhda” with peel
ε’	―	↓^1^ 25–85 cm^−1^	↓ 5–38 cm^−1^ ↑ 44–95 cm^−1^	↑ 4–120 cm^−1^
ε’’	―	↑ 6–8, 11–93, 116–120 cm^−1^	↑ 18–104 cm^−1^	↓ 73–120 cm^−1^
α, cm^−1^	―	↑ 11–95, 110–114 cm^−1^	↑ 20–104 cm^−1^	↓ 62–120 cm^−1^
n	―	↓84–105 cm^−1^	↑ 3–97 cm^−1^	↑ 3–100 cm ^1^

^1^ ↓ and ↑—significant decrease or increase in the parameter in the infected tuber relatively healthy, respectively. Frequency ranges where significant differences were observed are indicated next to the effect sign. “―”-No measurements were taken.

**Table 2:** Influence of fusarium disease on THz spectral characteristics in cereals of different species.

Parameter	Species and IDF/SDF Ratios ^1^		
	Barley *H.* *vulgare* ~4.5 ^2^	Wheat *T.* *aestivum* ~5.3	Oat *A.* *sativa* ~6.3
ε’	↑^3^ 3–120 cm^−1^	↓5–29 cm^−1^ ↑50–86 cm^−1^	↓3–51, 116–120 cm^−1^
ε’’	↑5–120 cm^−1^	↓16–96 cm^−1^	↓14–116 cm^−1^
α, cm^−1^	↑7–10, 39–117 cm^−1^	↓17–106 cm^−1^	↓15–96 cm^−1^
n	↑3–120 cm^−1^	↓5–96 cm^−1^	↓3–120 cm^−1^

^1^ IDF ― insoluble dietary fibers, SDF ― soluble dietary fibers. ^2^ IDF/SDF ratios were calculated on literature data [1,2,3,4,5,6,7,8,9,10,11,12,13,14,15,16,17,18,19,20,21,22,23,24,25,26,27,28,29,30,31,32,33,34,35,36,37,38,39,40,41,42,43,44]. ^3^ ↓ and ↑ ― significant decrease or increase in the parameter in the infected tuber relatively healthy, respectively. Frequency ranges where significant differences were observed are indicated next to the effect sign.

**Table 3:** Primers used to *Fusariun* sp. and *P. infestans* identification.

Species and Target	Primers (F and R)	Ref.
*F. graminearu* Intergenic Spacer of rDNA (IGS region)	5′-GTTGATGGGTAAAAGTGTG-3′ 5′-CTCTCATATACCCTCCG-3′	[5]
*F. avenaceum* gene translation elongation factor 1-alpha (*TEF1*)	5′-ATGGGTAAGGARGACAAGAC-3′ 5′-GGARGTACCAGTSATCATG-3′	[5,6]
*P. infestans* sites ITS1 and ITS2	5′-AACCCAATAGTTGGGGGTCTTAC-3′ 5′-TCGTCCCCACAGTATAATCAGTATTAA-3′	[7]

## Data Availability

The raw data supporting the conclusions of this article will be made available by the authors, without undue reservation.
